# An International Antibody Characterization Platform Enabling Alzheimer's Disease Research

**DOI:** 10.1002/alz70855_100417

**Published:** 2025-12-23

**Authors:** Riham Ayoubi, Aled M Edwards, Peter S McPherson, Carl Laflamme, Emory‐Sage‐SGC‐JAX TREAT‐AD Center, YCharOS initiative

**Affiliations:** ^1^ Montreal Neurological Institute‐McGill University, Montreal, QC, Canada; ^2^ University of Toronto, Toronto, ON, Canada

## Abstract

**Background:**

Understanding Alzheimer's disease (AD) etiology requires a comprehensive molecular and biological investigation of the estimated 184 genes/proteins linked to AD risk. However, most AD research focuses on 3 genes, APP, APOE, and TAU, which receive 49% of all AD‐related PubMed hits. This highlights the need for a broader exploration of AD risk factors. A major challenge in this regard is the limited availability of selective and renewable antibodies for studying all AD‐associated proteins.

The Target Enablement to Accelerate Therapy Development for Alzheimer's Disease (TREAT‐AD) program focuses on developing or identifying high‐quality research tools for less‐studied AD‐associated proteins, enabling hypothesis testing (Axtman et al., *Alzheimers Dement*, 2023).

**Method:**

Antibody Characterization through Open Science (YCharOS) is an international, public‐good initiative that unites academic researchers, funding agencies, leading antibody manufacturers and knockout cell line providers to evaluate antibody performance (Ayoubi et al., *Nature Protocols*, 2024). Using knockout cell lines as isogenic controls, antibodies from multiple manufacturers are tested side‐by‐side in western blot, immunoprecipitation and immunofluorescence. Results are disseminated openly and rapidly via the AD Knowledge Portal (https://adknowledgeportal.synapse.org/).

**Result:**

To date, 441 antibodies targeting 41 community‐prioritized AD targets have been characterized. Most cited antibodies for these proteins are polyclonal and often lack specificity, particularly in immunofluorescence experiments. However, our findings reveal that selective and renewable antibodies, many of which are newly generated and rarely used in published studies, were available for 90% of TREAT‐AD targets.

When all existing antibodies for a protein underperform, TREAT‐AD generates recombinant proteins as antigens for antibody development, collaborating with YCharOS partners for antibody production. This approach has been applied to AD‐related proteins, including SMOC1, yielding recombinant antibodies with superior selectivity over existing ones.

Using knockout‐validated antibodies, we demonstrated that the proteins CD44, Moesin, sFRP‐1 and Midkine, identified through systems biology by the TREAT‐AD initiative as having altered gene expression in AD, are upregulated under disease conditions in AD mouse models (Doolen et al. *F1000Research*, 2024).

**Conclusion:**

YCharOS has established characterization standards that align with funding and journal requirements, and is committed to identify reliable and accessible tools for AD research.

